# Idiopathic Transverse Myelitis and Neuromyelitis Optica: Clinical Profiles, Pathophysiology and Therapeutic Choices

**DOI:** 10.2174/157015911796557948

**Published:** 2011-09

**Authors:** Amer Awad, Olaf Stüve

**Affiliations:** 1 Department of Neurology, Case Western Reserve University, Cleveland, OH, USA; 2 Department of Neurology, University of Texas Southwestern Medical Center at Dallas, TX, USA; 3 Neurology Section, VA North Texas Health Care System, Medical Service, Dallas, TX, USA

**Keywords:** Transverse myelitis, neuromyelitis optica, epidemiology, pathology, pathogenesis, treatment.

## Abstract

Transverse myelitis is a focal inflammatory disorder of the spinal cord which may arise due to different etiologies. Transverse myelitis may be idiopathic or related/secondary to other diseases including infections, connective tissue disorders and other autoimmune diseases. It may be also associated with optic neuritis (neuromyelitis optica), which may precede transverse myelitis. In this manuscript we review the pathophysiology of different types of transverse myelitis and neuromyelitis optica and discuss diagnostic criteria for idiopathic transverse myelitis and risk of development of multiple sclerosis after an episode of transverse myelitis. We also discuss treatment options including corticosteroids, immunosuppressives and monoclonal antibodies, plasma exchange and intravenous immunoglobulins.

## INTRODUCTION

Multiple sclerosis is the prototype of inflammatory disorders affecting the central nervous system. However, there are numerous inflammatory conditions, other than multiple sclerosis that have the central nervous system as their only or preferred target (Table **1**). Early recognition of such syndromes is crucial for applying the appropriate therapy that can be quite different from that of multiple sclerosis. In this review we will focus the discussion on two important disorders closely related to multiple sclerosis: idiopathic transverse myelitis and neuromyelitis optica (Devic’s disease). We will briefly discuss the epidemiology and clinical presentations of these diseases and provide detailed discussion on the pathophysiology and therapeutic approaches. 

## IDIOPATHIC TRANSVERSE MYELITIS

Transverse myelitis (TM) is a focal inflammation of the spinal cord of different etiologies. It can be idiopathic or related/secondary to other diseases. The percentage of idiopathic TM is expected to be declining due to the advances in neurodiagnostics and the discovery of new disease markers. 

### Epidemiology and Clinical Profiles

The annual incidence of TM in the United States is around 4.6 per million per year [1]. The incidence of idiopathic TM is about 1.34-4.6 per million per year [2]. However, a study by Young and his co-workers found much higher numbers (3-5 times higher) in the Australian population [2]. TM has a bimodal distribution with two distinct peaks: 10-19 and 30-39 years. It shows no racial, familial or gender predilection [1, 3, 4]. About 28% of reported cases of TM are in pediatric population [5].

TM typically presents with acute to subacute myelopathy [6-12]. The symptoms usually progress over hours to few weeks. The thoracic cord is the most common to be affected for no clear reasons. Many patients present with flu-like symptoms prior to the myelopathy picture. The most common symptoms include: back pain (30-50%), lower limb paresthesias (80-95%), allodynia (80%), paraparesis (50%), bladder symptoms (almost 100%) and sensory level (80%). 

**Fig. (1):**
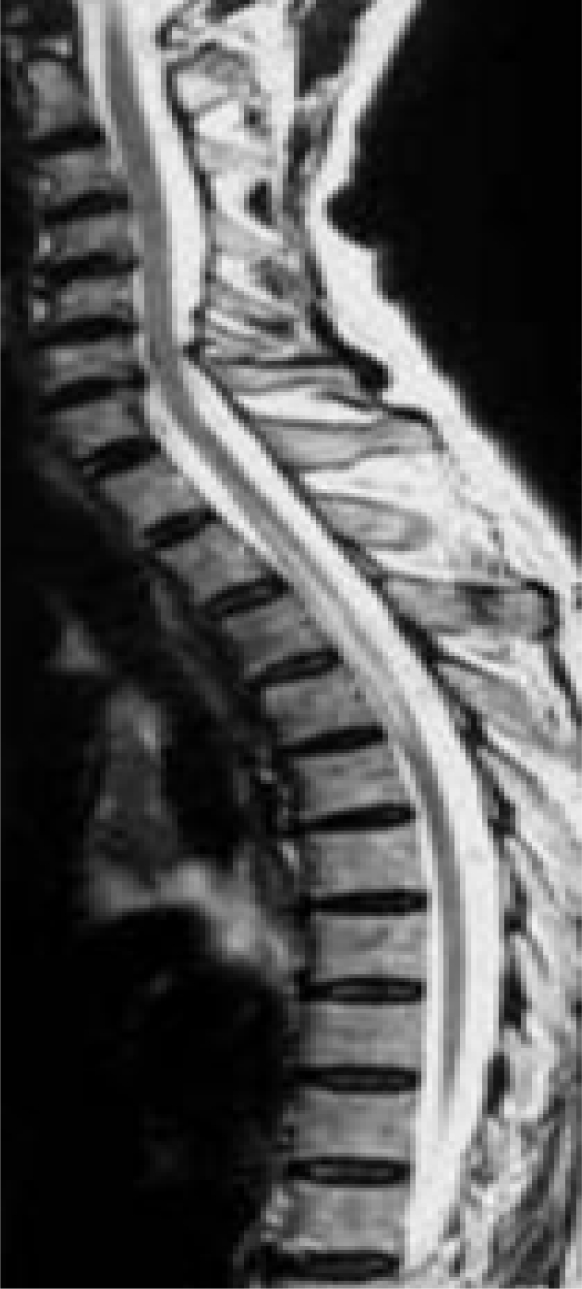
T2 Sagittal spine MRI of a 30 year old lady presented to our hospital with left lower extremity weakness and low back pain showing the typical fusiform cord signal in TM.

The idiopathic TM proposed diagnostic criteria are shown in Table **2** [13]. Abnormal cord signal on spine magnetic resonance imaging (MRI) (Fig. **1**) can be seen in around 75% (50-90%) [11, 14-17]. The cerebrospinal fluid (CSF) shows nonspecific signs of inflammation like elevated protein level and pleocytosis in about one half of adult patients, [1, 8, 17, 18] and about 80% of children with TM [14]. Oligoclonal bands in CSF are typically absent in non-MS related TM and if present are usually transient [17, 19]. TM is typically monophasic but relapsing TM does occur in about 20-30% [20-22]. Male gender, strict white matter involvement and normal CSF parameters seem to increase the risk of recurrence [23]. 

The most important findings that increase the chance of progression to MS include: abnormal brain MRI, incomplete (partial) iTM, the presence of oligoclonal bands in the CSF, abnormal visual and somatosensory evoked potentials and human leukocyte antigen (HLA) DR-2 status [2, 8, 24-28]. The rate of conversion to MS in patients with iTM with brain lesions ranges is 44-93% and without brain lesions is 15-44% [2]. Oligoclonal bands in CSF are frequent with iTM (~62% vs. 17%) [2].

Poor prognosticators include: back pain, rapid progression, cervical spine involvement, spinal shock, denervation on electromyography (EMG) and the presence of protein 14-3-3 in the CSF [9, 15, 29-31]. 

### Pathology, Pathogenesis and Pathophysiology

Most of information on TM pathology comes from clinicopathological cases reports and autopsy studies [32-34]. All patients who met the criteria for TM had inflammation. The lesions in acute TM are invariably limited to the spinal cord with no involvement of other structures in the central nervous system (CNS). The histopathology is characterized by demyelination and axonal loss in addition to gray matter involvement. Necrosis and cavitation can result in severe cases. The cord involvement is usually central, uniform and symmetric in comparison to multiple sclerosis which typically affects the cord in a patchy way and the lesions are usually peripheral. Perivascular spread of monocytes; lymphocytes infiltrating focal areas of the cord along with astroglial and microglial activation are invariable findings on histopathology of TM. In some biopsies during the acute phases of TM, infiltrates of CD4^+^ and CD8^+^ T-lymphocytes were found to be prominent suggesting immune-mediated disease process. There is typically preservation of the subpial parenchyma suggesting ischemia as the ultimate cause of the cord lesions in TM. The pathology also differs depending on the etiology. For example necrotizing myelitis can be seen in NMO (see below) and paraneoplastic myelitis [35]. In MS and acute disseminated encephalomyelitis (ADEM) the lesions tend to have predilection to the white matter in comparison to the circumferential involvement in idiopathic TM [36]. 

Various infections precede 30-60% of the cases of TM [1, 3, 4, 7-9, 11, 37]. Reported infections include, but not limited to: herpesviridae, enteroviruses, influenza viruses, adenoviruses; coxsackie viruses; enteric cytopathogenic human orphan (ECHO) virus, hepatitis A virus; lymphocytic choriomeningitis virus (LCMV); mumps virus; measles virus, rubella virus, poliovirus, rubeola virus, dengue virus, Russian spring-summer encephalitis virus, varicella virus, mycoplasma pneumonia bacteria, legionella pneumonia bacteria, pulmonary tuberculosis, borrelia (Lyme disease), listeria, and bartonella (cat scratch disease) [4, 11, 38, 39]. About 30% of pediatric cases are preceded with immunizations within one month of disease onset [7, 11].

Infections can cause TM through direct tissue damage, [39-41] or by immune-mediated infection-triggered tissue damage which may be due to molecular mimicry or superantigen effect. The molecular mimicry theory is based on the fact that several infectious agents are capable of encoding molecular structures (e.g. proteins, glycolipids or proteoglycans) that mimic self antigens [4, 42-44]. Immune response to the mimic “pseudo-self” then may result in cross-reactive immune activation against self tissue. The immune response can be either T-cell mediated or antibody mediated. Superantigens are microbial peptides that are capable of inducing fulminant immune response by activating a large number of lymphocytes including autoreactive T- cells in a unique fashion by binding to the Vβ region of T cell receptor instead of highly variable peptide groove [45-49]. Superantigens are also capable of activating T- lymphocytes in the total absence of co-stimulatory molecules.

A non-microbial related immune dysfunction has been also proposed in the immunopathogenesis of TM. Some studies have described the presence of autoantibodies in TM [18, 22, 50].Interleukin 6 (IL-6) levels were also markedly elevated in the spinal fluid of TM patients in comparison to control patients and to MS patients, and this also correlated with disability [4, 51]. IL-6 is secreted by astrocytes and microglia and binds to oligodendroglia and axons. High levels of IL-6 can cause direct tissue injury and indirect damage by inducing nitric oxide synthetase in microglia. Interestingly, Interferon-beta (IFN-β), a medication used to treat MS, was found to induce IL-6 [52]. IL-6 has probably a bell-shaped effect where at certain levels could cause damage and at different levels can induce repair [51, 53, 54]. 

One study that was conducted in Japan found that several patients with TM have much higher serum IgE levels than MS patients or controls pointing towards immune-mediated process as well [55]. Concordant with these findings, tissue biopsies of two patients with TM and elevated total and specific serum IgE disclosed the presence of antibody deposition within the spinal cord and perivascular infiltration with eosinophils that could induce tissue damage [56]. 

### Current Therapies 

The main therapies available for TM include: high dose intravenous methylprednisolone (IVMP), plasma exchange and/or intravenous cyclophosphamide. There are no placebo-controlled randomized trials to support the use of IVMP in TM despite the positive clinical experience. However, there are several small observational studies that documented clinical benefit of IVMP in TM, if given in the acute phase, in terms of faster recovery and disability in addition to good tolerability [57-62]. The rationale of using steroids in TM is based on its numerous effects on the immune system leading to a global immunosuppression. Some of these effects include but not limited to: inhibition of lymphocyte proliferation and differentiation, redistribution of lymphocytes, alteration of lymphokine function especially tumor necrosis factor (TNF), IL-1 and IL-2, and inhibition of macrophage function, in particular antigen presentation and processing. 

In comparison to steroids, use of plasma exchange in TM is supported by randomized trials. It is commonly initiated in severe cases of TM and in cases unresponsive to pulse steroids. Plasma exchange is an effective way to remove “autoreactive antibodies” and other toxic molecules from the plasma and hence, it is a very common procedure in the acute and rescue treatment of autoimmune diseases. Several studies have shown significant clinical benefit for plasma exchange in TM [63-66]. Based on the NMO literature (see below), early initiation of plasma exchange within 20 days of attack onset predicts greater likelihood of clinical response [65].

There is currently no solid evidence to support the use of immunosuppressive therapy in TM. Cyclophosphamide, an alkylating agent that showed some promising results in MS can be used in idiopathic TM, especially in severe cases [67]. A retrospective study of 13 patients with idiopathic TM, intravenous cyclophosphamide in combination with plasma exchange was shown to be superior to plasma exchange alone in patients with complete TM [66]. Cyclophosphamide, in addition to its antimitotic effect, has significant immunosuppressive and immunomodulatory actions making it an effective treatment for immune-mediated disorders. It causes suppression of cell-mediated and humoral immunity through its actions on T cells and B cells [68]. In multiple sclerosis, for example, cyclophosphamide was shown to decrease the secretion of the pro-inflammatory cytokines like IFN-γ and IL-12 and increase the secretion of the anti-inflammatory cytokines like IL-4 and IL-10 in cerebrospinal fluid (CSF) and peripheral blood [69]. However, due to the significant side effects like gonadotoxicity and bladder toxicity its use should be individualized and carefully initiated and monitored. Other oral immunosuppressive agents like mycophenolate, azathioprine and methotrexate can also be considered in recurrent cases despite the lack of evidence. In our clinical experience we use IVMP alone if the patient presents with non-motor symptoms, plasma exchange in patients presenting with incomplete TM with motor symptoms and cyclophosphamide if the patient presents with complete TM. Cyclophosphamide, in our experience is a well-tolerated and a relatively safe medication if used appropriately and in the right clinical setting. We discussed cyclophosphamide in great details in a separate review that was recently published in 2009 [67].

Interferon-β (IFN-β) and glatiramer acetate (GA) have a well-established role in MS and they are FDA-approved for the treatment of MS. They also have well-documented benefit in clinically isolated syndromes *with brain lesions* [70-73]. In conclusion, IFN-β and GA would be ideal choices in patients with TM that carry a high risk of conversion to MS. 

The ultimate therapy of TM would be targeted therapy once the targets are clearly defined. Like in all neurological disorders, the search for the ideal neuroprotection that can be used in combination with the treatments mentioned above probably provides the most practical treatment strategy at this time. The use of erythropoietin in TM is under investigation. Erythropoietin was shown to be neuroprotective in different models of nerve injury in animal studies, [74, 75] and clinical studies are planned to investigate the safety, tolerability and efficacy of erythropoietin in TM patients. 

## NEUROMYELITIS OPTICA SPECTRUM (DEVIC’S DISEASE)

Neuromyelitis optica (NMO) is an idiopathic, probably immune-mediated, destructive demyelinating syndrome of the CNS with predilection to optic nerves and spinal cord. 

### Epidemiology and Clinical Profiles 

NMO has a significant female to male predominance (9:1), and affects mainly young people with a median age of onset in the late 30’s [76-78]. In the United States it mainly affects Caucasians but the other racial groups are significantly afflicted in comparison to MS [76, 77, 79-81]. NMO does occur in children with an average age of 10 years (7-14 years) [82, 83]. Opticospinal MS (OSMS), which is *controversially* recognized as part of NMO spectrum, is prevalent in Japan [84]. NMO may account for 1% of inflammatory demyelinating disease (IDD) of the CNS among Caucasians, but represents almost 30% of total IDD in Japan [85].

Familial clusters of NMO have been described in scattered reports in the literature with no conclusive evidence [86, 87].

NMO is quite different from MS clinically, radiologically, pathologically and in terms of treatment approaches. The distinction is of paramount importance because management and prognosis are fundamentally different. Clinical onset is usually acute and in two thirds of cases, a prodrome of flu-like symptoms may precede neurologic deficits [82, 88]. Various viral and non-viral infectious have been seen to precede neurologic symptoms of NMO with no conclusive association [89-96].

The first manifestation of neuromyelitis optica is ON in up to three fourths of patients, TM in up to one third and concomitant ON and TM in up to one tenth of patients [88, 97]. The interval between visual and cord symptoms is extremely variable, ranging between days to years [88, 98, 99]. ON is unilateral in 65-85%, but bilateral simultaneous ON or sequential ON in rapid succession is more suggestive of NMO [100]. NMO-ON clinical features are similar to MS-related ON but more severe leading to complete visual loss in 40% of patients [101]. In 5 years, more than half of patients with relapsing NMO are blind in one or both eyes or require ambulatory help [100]. NMO-TM is usually severe, as well. It is typically longitudinally extensive TM (LETM); extending over 3 or more cord signals [101]. The spinal cord inflammation is usually symmetric causing symmetric involvement of motor and sensory tracts [101]. Cervical LETM in NMO may extend into the medulla resulting in nausea, persistent hiccups, and even fulminant respiratory failure [88, 102]. Lhermitte sign, tonic spasms and radicular pain are reported in one third of NMO patients [101, 103]. Lhermitte sign and the paroxysmal tonic spasm indicate a relapsing more than a monophasic course [101].

NMO is monophasic in 10-30% of cases and relapsing 60-90% of cases [101]. As mentioned above, NMO ON and TM attacks are typically more severe and recovery is less complete compared with MS [100].The relapse rate is higher than in MS, frequency of a second episode within the first 2 years is also higher than in MS ( 82% vs. 62%) and the median time to reach expanded disability status scale (EDSS) of 6.0 is shorter than MS( 7.0 years vs. 9.4 years) [97]. Relapses occur within 6 months of the initial attack in 55% of patients, within 18 months in 27%, and after 5 years in 18% [88]. The mean interval until a second attack is about 17 months [97]. In comparison to adults, children with NMO have fewer relapses [82, 104]. 

In contrast to MS, secondary progression is quite rare and progression only takes place during the attack and disability is a result of the clinical cumulative effect of relapses rather than secondary progression [100]. The monophasic form has a better prognosis than the relapsing form [97-99, 101]. The survival rate at 5 years may amount to 90% in the monophasic groups and 68% in the relapsing group. Poor outcome and mortality are directly related to high relapse rate during the first 2 years [97, 100, 105].

**Fig. (2a, b):**
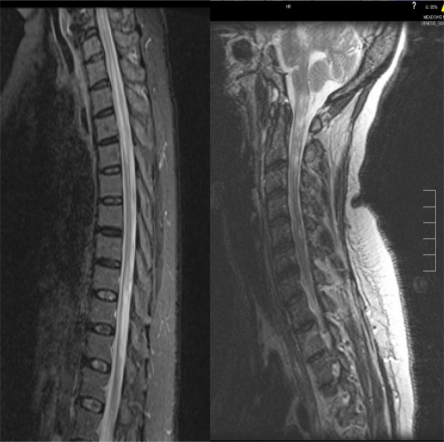
Fig. (**2a**) shows a T2 sagittal spine MRI of a 38 year-old woman who presented to our hospital with acute incomplete paraparesis displaying the typical LETM in acute NMO. Fig. (**2b**) shows a T2 sagittal spine MRI of a 51 year-old woman with chronic relapsing NMO showing the typical manifestations of chronic NMO including cord atrophy and patchy lesions.

MR imaging of the spine is very helpful in diagnosing NMO. In the acute phase it typically shows LETM with a T2 cord signal with cord expansion and gadolinium enhancement (Fig. **2a**) [106]. In the chronic stage the enhancement and expansion resolve while the T2 signal persists but the LETM regresses to less than 3 segments and breaks up into shorter fragments and the cord becomes atrophic (Fig. **2b**) [106]. Up to 60% of NMO patients will develop non-specific asymptomatic subcortical white matter lesions at a mean follow up of 6+/-5 years [107]. Ten percent of patients have specific lesions in the diencephalon (thalamus, hypothalamus), corpus callosum, hypophysis, brainstem (periaqueductal) and around 10% of NMO patients will develop lesions that meet the specific MS criteria [107-109]. Magnetization transfer (MT) and diffusion tensor imaging (DTI) disclosed microscopic pathology in the normally appearing gray matter (NAGM) in the brain of patients with NMO [110]. 

Optical coherence tomography (OCT) is a relatively new noninvasive method of evaluating the thickness of the retinal nerve fiber layer (RNFL). The mean retinal nerve fiber layer thickness is significantly reduced in patients with NMO compared with controls and has good correlation with EDSS [111]. Findings on OCT could potentially differentiate NMO ON vs. MS ON. NMO ON is associated with a thinner mean RNFL compared to MS and the superior and inferior quadrants were more severely affected in NMO than MS [112-114].

In terms of CSF studies, NMO profile is quite distinct from MS. The white blood cell count (WBC) is usually 50-1000 cells/mm3 vs. 0-50 in MS, shows neutrophilic predominance vs. lymphocytic predominance in MS [101]. CSF pleocytosis is more prominent during the acute attack of TM more than ON and has a specificity of 95% and sensitivity of less than 30% [101]. Patients with severe TM accompanied by marked CSF pleocytosis (more than 100 leukocytes/mm3) have typically poor outcome [88]. CSF oligoclonal bands are seen in 15-30% in NMO vs. 85% in MS and have low sensitivity and specificity in NMO [101]. An elevated IgG index is only seen in 20% of NMO patients [101]. Up to one half of patients with NMO have positive serological immunological markers like (antinuclear antibody (ANA), extractable nuclear antigens (ENAs), anti-thyroid antibodies, anti-cardiolipin antibodies, anti-parietal cell antibodies and acetylcholine receptor antibodies [101, 115-120]. 

Seropositivity for Sjögren’s’ syndrome (SS)-A (anti-Ro) antibody is modestly predictive of relapse in patients with the first TM attack, seen in 77% of relapsing NMO vs. 33% of monophasic NMO [121]. For relapsing NMO, the odds ratio for having another autoimmune disease is increased by 10-fold [100]. Risk factors for recurrence include: NMO-IgG seropositivity, female gender, older age (>30 years), *less* severe motor impairment after the myelitic onset, longer interval between the first and second attack (>6 months) and systemic autoimmunity [88, 100, 101]. 

Diagnostic Criteria for NMO were initially proposed in 1999 [101]. However, the discovery of NMO-IgG anti-body [122] revolutionized the diagnosis of NMO and the criteria were revised in 2006 (Table **3**) [84]. In a cohort of patients examined between 1999 and 2005, the new criteria were 99% sensitive and 90% specific for neuromyelitis optica, compared to the previous criteria, which were 85% sensitive and 48% specific [123]. In summary, NMO antibody and MRI spine are the most important diagnostic tools for NMO. 

The discovery of NMO antibody has revolutionized the accuracy of NMO diagnosis and broadened the spectrum of NMO syndromes more than previously appreciated but its role in pathogenesis is controversial and unclear. NMO antibody was discovered by Lennon and co-workers 2004 [122]. Its sensitivity ranges from 58-76% and specificity ranges from 85- 99% [84, 122, 124, 125]. In a large study comparing the sensitivity and specificity of immunofluorescence (IF) and immunoprecipitation (IP) assays using green fluorescent protein-tagged Aquaporin-4 (AQP4), the sensitivity rates for NMO were higher using IF technique and improved by 5% using combined methods [126]. NMO antibody is also seen in syndromes closely related to NMO, for example: recurrent LETM (52%), recurrent isolated ON (25%), and the Japanese form of optic-spinal MS (58%) which expands NMO to syndromes rather than one disease though the associations are still debatable and not completely understood [122]. Its target antigen is the water channel aquaporin-4 (AQP-4). Aquaporins are involved in fluid balance and cell water homeostasis and AQP-4 is the most common type found in the CNS specifically on astrocytes foot processes, the abluminal surface of blood vessels (glia limitans) and concentrated in the periependymal regions of the brain [127-129]. We will discuss the role of NMO antibody in NMO pathogenesis in a separate section. In conclusion, NMO is a very specific marker that defines the NMO spectrum of disorders: complete NMO and incompletely developed (restricted) type of NMO [77, 122, 130]. Its role in pathogenesis is unclear. It was shown also to predict a relapsing course and a poor outcome in some studies [123, 131]. 

### Pathology, Pathogenesis and Pathophysiology 

Spinal cord specimens from patients with NMO typically show evidence of perivascular inflammatory demyelination with high proportion of polymorphonuclear cells (PMN) and eosinophils, deposition of IgM and complement C9 neoantigen in addition to hyalinization of spinal cord arteries with gray and white matter necrosis [102, 129, 132-135]. Contrary to MS lesions, there is rare infiltration with CD3 ^+ ^and CD8^+ ^lymphocytes [85]. Prominent vasculocentric IgG and complement deposits, with vascular proliferation and fibrosis are also typical findings in NMO and correspond to AQP-4 site of expression and might be related to humoral immune activation and a pathogenic NMO-IgG. The anterior optic pathway shows significant demyelination and cavitation in NMO specimens as well [98]. 

The etiology of neuromyelitis optica remains unknown. A possible role for autoimmunity in the pathogenesis is suggested by several observations. The overlap with other autoimmune diseases and the wide range of positive autoantibodies in the serum of patients with NMO, as mentioned above, suggests polycloncal B cell activation [132]. The discovery of the specific NMO antibody supports the autoimmune theory as well. The role of infections in NMO pathogenesis is not clear, but the various infections that may precede NMO neurological symptoms could theoretically induce immune dysregulation (see the section on infections and TM). The immunohistological findings of prominent infiltration with neutrophils, eosinophils, and IgM deposition in the lesions also support the immunological theory, especially the significant involvement of humoral immunity in the immunopathogenesis of NMO. A recent molecular study by Pentón-Rol and co-workers found that the levels of IL-10 and tumor necrosis alpha (TNF-α) were undetectable pointing to immunoregulatory dysfunction [136]. The good clinical response to immunomodulatory treatments (see below) also supports the immune theory. Besides, increased number of myelin oligodendrocyte glycoprotein-specific B cells was also demonstrated in NMO pointing towards dysregulation of humoral immunity [137]. Very interestingly, several reports have described elevated prolactin levels in some patients with NMO spectrum [108, 138, 139]. The elevated prolactin levels were seen mostly in Asian and black women and was concordant with the presence of ON. The elevated prolactin could be a result or a contributing factor. The extension of inflammation to involve the hypothalamus could result in disinhibition of prolactin release. Prolactin, on the other hand, is a potent immune stimulant for T helper cell 1 (T_H_1) cell responses that could be involved in the initiation or sustenance of the neuroinflammation [140]. Of note, elevated prolactin levels is not a unique finding to NMO and there are numerous reports of hyperprolactinemia in other autoimmune diseases especially SLE [141, 142].

The role of genetic factors in NMO is not determined yet. There is evidence, however, that NMO is associated with HLA alleles DR1*801, DP1 501, DPA1 202, DPB1*1501, DRB*03 but not with DRB1*1501 or DRB5*0101, which are classically associated with MS [143-146]. These findings may suggest a possible genetic role in the disease process.

The role of NMO Ig-G antibody in the immunopathogenesis of NMO is quite complicated and not well-understood. The observation that brain lesions in NMO occur in areas with high concentration of AQP-4 brought the attention to the role of the anti-AQP-4 antibody in the disease process [107]. However, there are several observations that argue against the pathogenic role of the antibody. Firstly, The brain lesions are not really the sine qua none of NMO and they’re usually clinically insignificant despite the high density of AQP-4 in the brain. In addition, AQP-4 is present in the kidney and gastrointestinal tract, but involvement of these regions has not been described in neuromyelitis optica so far and AQP-4 does not explain the distribution of NMO lesions. Pathological studies have failed to demonstrate the presence of NMO antibody in NMO lesions [147, 148].

The NMO experience with animal models is very interesting. When human NMO-IgG antibody was given to rats, NMO pathology could not be reproduced, but when it was injected into animals induced for experimental auto-immune encephalomyelitis (EAE), NMO lesions were then reproduced. The conclusion that can be drawn from these findings is that a disruption of blood brain barrier may facilitate the pathogenic role (if any) of NMO antibody. The bottom line is that the pathogenic role of the AQP-4 antibody is unknown and needs to be studied more carefully before drawing any solid conclusion. 

The relation between NMO and OSMS is controversial and debatable. For example, several reports argued that OSMS is quite distant from NMO from a pathologic point of view [132, 149, 150]. The immunologic studies found that OSMS is mostly T helper cell 1 (T_H_1)-related disease in comparison to NMO which is thought to be T helper cell 2 (T_H_2) and B-lymphocyte-driven making them completely different diseases. Further studies are needed to clarify the relation between NMO and OSMS. 

### Curent Therapies (Table 4)

There are currently no FDA-approved medications for NMO due to the lack of large double-blinded randomized placebo-controlled trials. 

Intravenous pulse steroids (IVMP 1 gram IV every 24 hours for 5 days followed by oral steroid taper is the usual treatment of acute attacks of NMO [151]. The immunological effects of steroids are discussed in the TM section. Rescue treatment with plasma exchange is supported by several clinical trials [63, 65, 152]. Early initiation of plasma exchange within 20 days of attack onset predicts greater likelihood of clinical response [65]. There are few case reports of lymphocytapheresis as being effective in NMO as well [153]. Plasma exchange and lymphocytapheresis aim at removing the “pathogenic” factors from plasma as mentioned above in the TM section. 

The most promising treatment for relapsing NMO is probably rituximab. Rituximab is a monoclonal antibody that targets cluster of differentiation (CD) 20+ cells but its exact mode of action remains unclear [154]. Possible actions may include: induction of antibody-dependent cell cytotoxicity (ADCC) and complement-dependent cytotoxicity, regulatory effects on cell cycle, increase antigen presentation, downregulation of B-cell receptors and induction of B-cell apoptosis. An open label study of the effects of rituximab in NMO showed that 6/8 patients with worsening NMO refractory to prior therapy, treated with IV rituximab, remained relapse free for a 6-18- month-follow up and the median attack rate declined from 2.6 to 0 attacks per patient per year (p=0.0078) [155]. The typical dose used is 1000mg (1gm) or 375mg/m2 IV infusion followed by monthly monitoring of CD19 count and once CD19 levels are detectable, rituximab is redosed [155]. Side effects of rituximab may include but not limited to: infusion-related reactions, increased risk for reactivation of tuberculosis (TB), hepatitis B virus (HBV) and JC virus [155]. Jacob and co-workers conducted a retrospective analysis of 25 patients with NMO of whom 23 had frequent relapses despite the use of medical therapy [156]. Infusions of rituximab at median intervals of 8 months decreased the median relapse rate after a median follow up of 19 months (0-3.2 (post-treatment) vs. 0.5-5 (pre-treatment) relapses, P <.001). Very recently, a trial of rituximab in 3 patients with NMO showed significant reduction of relapse rate [157]. The annualized relapse rate for the 3 patients during the year before rituximab therapy was 4, 5, and 6, respectively, and this decreased to 3, 1, and 0 in the year after therapy. In our clinical experience, we use one induction dose of rituximab and then we follow the CD19 counts and redose once the CD19 is detectable (≥1%), typically every 6-9 months for ≤ 2 years. Weekly infusions of rituximab for 4 weeks as induction protocol have been used especially in treating rheumatologic disorders like refractory rheumatoid arthritis [158]. The natural course of the disease in NMO mandates *aggressive* and *emergent* treatment because the attacks carry a high risk of disability and in some cases the attacks can be fatal. It cannot be overemphasized that NMO should be treated aggressively since we have a very small room for trial and error. Studies comparing the efficacy and safety of different dosing regimens are needed to unify our approach in treating these patients. 

In our experience, if a patient relapses while sufficiently treated with rituximab, we treat the acute exacerbation with high dose IV steroids in addition to plasma exchange and IV cyclophosphamide in refractory cases. After stabilizing the patient we would switch the patient to IV cyclophosphamide.

Other immunomodulatory therapies that have showed good results in NMO include: azathioprine, mitoxantrone, methotrexate, mycophenolate, cyclophosphamide and intravenous immunoglobulin (IVIG). Azathioprine (2-3 mg/kg/day) in combination with oral prednisone (1mg/kg/day) was used in an observational case series of 7 patients was shown to be effective in prevention of relapses in NMO [159]. The drug is a purine analog which is metabolized to 6-meraptopurine (MP) and ultimately, becomes incorporated into replicating DNA and can also block the de novo pathway of purine synthesis [160]. Its relative specificity to lymphocytes is explained by the lack of the salvage pathway of purine synthesis [160]. Mitoxantrone was also shown to be helpful in NMO [161]. Mitoxantrone is as a doxorubicin analogue that modulates the immune system by several mechanisms. It suppresses the proliferation of T cells, B cells, and macrophages, impairs antigen presentation and decreases the secretion of proinflammatory cytokines, enhances T-cell suppressor function and inhibits B-cell function and antibody production and inhibits macrophage-mediated myelin degradation [162]. Methotrexate was also tried in NMO and showed some benefit. Methotrexate, at lower doses, has been shown to be very effective for the management of many autoimmune disease due the inhibition of enzymes involved in purine metabolism, leading to accumulation of adenosine, or the inhibition of T cell activation and suppression of intercellular adhesion molecule expression by T cells [163]. Mycophenolate is another immunosuppressant that has been used in NMO patients. It was found to decrease relapse rate in a retrospective trial of 24 patients with NMO spectrum [164]. Mycophenolate mofetil is metabolized in the liver to the active moiety mycophenolic acid which then inhibits inosine monophosphate dehydrogenase, the enzyme that controls the rate of synthesis of guanine monophosphate in the de novo pathway of purine synthesis used in the proliferation of B and T lymphocytes [165]. As mentioned above, the lack of salvage pathway in lymphocytes renders them targets for the action of inhibitors of de novo pathway of purine synthesis. Immunoablative doses of cyclophosphamide were shown to control disease progression in intractable cases of NMO [166]. Immunological mechanisms of action of cyclophosphamide are described in the TM section. Using immunoablative doses of cyclophosphamide is an intriguing and relatively novel approach that had very promising results in MS as well [67]. Repeated IVIG infusions were also used in 2 patients with NMO and showed some positive results [167]. The exact mechanism by which IVIG suppresses inflammation has not been definitively established but is believed to involve the inhibitory Fc receptor which can lead to decreased antigen presentation [168]. IVIG may work *via* inducing an immune complex formation that binds Fc receptors on dendritic cells that probably mediate its anti-inflammatory effects [169]. It may also work by directly binding the pathogenic antibodies or *via* complement activation “complement scavenging effect” [170]. IVIG also reacts with a number of membrane receptors on T cells, B cells, and monocytes that are pertinent to autoreactivity and induction of tolerance to self [171]. A recent report stated that IVIG application to activated T cells leads to their decreased ability to engage microglia [172]. 

Immunomodulators that are typically used in treating MS like IFN-β were found to be ineffective and even harmful in patients with NMO based on small trials [173]. Up to our knowledge, there is only one reported case of NMO that responded to GA [174]. Making decisions on single reported cases can be done but with extreme caution. Based on the current evidence, we cannot recommend using IFN-β and GA in the treatment of NMO. Since recovery is usually incomplete and the attack is more debilitating than in MS, there is a small room for trial and error when it comes to treatment of NMO and only the most effective treatment should be offered. 

As we mentioned in the TM section, future therapeutic approaches should focus on neuroprotection and targeted therapy. There no ongoing clinical trials, up to our knowledge, studying neuroprotection in NMO.

## Figures and Tables

**Table 1:** Inflammatory Disorders Related to Multiple Sclerosis

Clinically isolated syndromes (optic neuritis, transverse myelitis) Neuromyelitis optic (Devic’s disease) Acute disseminated encephalomyelitis (ADEM) Bickerstaff’s brain stem encephalitis Neuro-Bahçet’s disease Neurosarcoidosis Neuro-Sjogren’s disease Systemic lupus erythematosus (SLE) Inflammatory ocular diseases Central nervous system vasculitis Arachnoiditis Paraneoplastic encephalitic syndromes Steroid-responsive encephalopathy (Hashimoto’s encephalopathy) Infections ( Inflammatory form of progressive multifocal leukoencephalopathy (PML), neurosyphilis, Whipple’s disease, human T-cell lymphoma-leukemia virus (HTLV-1), neuroberreliosis (Lyme’s disease), human immunodeficiency virus (HIV), neurobrucellosis, human herpes virus-6 (HHV-6), mycoplasma, subacute sclerosing panencephalitis (SSPE)

**Table 2:** Proposed Diagnostic Criteria for Acute Idiopathic Transverse Myelitis

**Inclusion criteria**
Development of sensory, motor or autonomic dysfunction attributable to the spinal cord
Bilateral signs and/or symptoms (though not necessarily symmetric)
Clearly-defined sensory level
Exclusion of extra-axial compressive etiology by neuroimaging (magnetic resonance imaging, or myelography; computerized tomography of spine not adequate)
Inflammation within the spinal cord demonstrated by cerebrospinal fluid pleocytosis *or* Elevated IgG index *or* gadolinium enhancement. If none of the inflammatory criteria is met at symptom onset, repeat MRI and LP evaluation between 2-7 days following symptom onset meets criteria
Progression to nadir between 4 hours to 21 days following the onset of symptoms (if patient awakens with symptoms, symptoms must become more pronounced from point of awakening)
**Exclusion criteria**
History of previous radiation to the spine within the past 10 years
Clear arterial distribution clinical deficit consistent with thrombosis of the anterior spinal artery
Abnormal flow voids on the surface of the spinal cord consistent with arteriovenous malformation
*Serologic or clinical evidence of connective tissue disease (sarcoidosis, Bahçet’s disease, Sjogren’s syndrome, systemic lupus erythematosus, mixed connective tissue disorder etc)
*Central nervous system manifestations of syphilis, Lyme disease, human immunodeficiency virus (HIV), human T-cell lymphoma-leukemia virus(HTLV-1), mycoplasma, other viral infection (e.g. herpesviridae viruses , enteroviruses)
*Brain magnetic resonance imaging abnormalities suggestive of multiple sclerosis
*History of clinically apparent optic neuritis
* Do not exclude disease-associated acute transverse myelitis

**Table 3:** Revised Diagnostic Criteria for Definitive NMO

Optic neuritis Acute myelitis Two out of three supportive criteria that include: Continuous spinal cord MRI lesion extending over 3 or more vertebral segments Brain MRI not meeting diagnostic criteria for MS NMO-seropositive status

**Table 4:** Management of Neuromyelitis Optica

Medication	Use	Typical dose	Evidence
High dose IV methylprednisolone	Acute	1 gm IV daily for 5 days with or without a taper	Observational studies
Plasma exchange	Acute as a rescue therapy	5 exchanges (each exchange 250 ml) over 5-10 days	Randomized trials in TM patients
Rituximab	Maintenance	1 gm (or 375 mg/m^2^) IV every 1-2 weeks for 2-4 weeks then redoes based on CD19 count (typically every 6-8 month) for ≤ 2 years	Several open label and retrospective clinical trials
Azathioprine	Maintenance	2 mg/kg PO divided BID (typically 100 mg BID) for ≤2 years	Observational studies
Mycophenolate	Maintenance	1-3 gm PO daily divided BID or TID for ≤2 years	Retrospective trial
Methotrexate	Maintenance	5-15 mg PO weekly for ≤2 years	Open label trial
Mitoxantrone	Maintenance	12 mg/m^2 ^every 3 months (maximum dose 140 mg/m^2^)	Open label trial
Cyclophosphamide	Maintenance	0.5-1.5 mg/m^2^ (typically 1 gm) IV every month until absolute lymphocyte count<1000/mm^3 ^(typically 6 cycles) or immunoablative dose of 200 mg/kg divided over 4 days	Open label trial
IVIG	Maintenance	2 gm/kg induction followed by 0.4-0.5 gm/kg every month	Case series
